# Tracking reduction-induced molecular changes in pathological free light chains by SV-AUC

**DOI:** 10.1007/s00249-025-01788-2

**Published:** 2025-08-05

**Authors:** Florian T. Tucholski, Rebecca Sternke-Hoffmann, Thomas Pauly, Rasmus K. Norrild, Amelie Boquoi, Roland Fenk, Luitgard Nagel, Alexander K. Buell, Rainer Haas, Dieter Willbold

**Affiliations:** 1 Institut für Physikalische Biologie, Heinrich Heine University, Düsseldorf, Germany; 2 PSI Center for Life Sciences, 5232 Villigen, Switzerland; 3 Institute of Biological Information Processing (IBI-7: Structural Biochemistry), Research Center Jülich, Jülich, Germany; 4 Protein Biophysics Group, Department of Biotechnology and Biomedicine, Technical University of Denmark, Søltofts Plads, Building 227, Kgs., Lyngby, Denmark; 5 Department of Hematology, Oncology and Clinical Oncology, Heinrich Heine University, Düsseldorf, Germany; 6 Department of Hematology and Stem Cell Transplantation, West German Cancer Center and German Cancer Consortium, University Hospital Essen, University of Duisburg-Essen, Essen, Germany

**Keywords:** Analytical ultracentrifugation, Patient derived immunoglobulin free light chains, Multiple myeloma, Monomer–dimer distribution, Aggregation

## Abstract

Multiple myeloma is a blood cancer characterized by plasma cell proliferation and excessive production of monoclonal proteins, often leading to renal complications and other forms of organ damage. A set of nine immunoglobulin free light chain (FLC) samples purified from urine of multiple myeloma patients was subjected to sedimentation velocity analysis. Aim of the study was to track changes of the oligomerization state of each FLC while triggering reduction-induced aggregation into larger structures. Sedimentation velocity experiments, combined with further techniques sensitive to structural changes, were performed to determine the degree of FLC oligomerization in each patient sample under different experimental conditions. Structurally, the FLC monomers are stabilized by two intramolecular disulfide bonds, while covalent dimerization occurs through an unpaired C-terminal cysteine residue. Incubation with the reducing agent TCEP cleaves intra- and intermolecular disulfide bonds, destabilizing both monomers and dimers. Remarkably, different incubation times revealed that destabilized dimers do not dissociate into stable monomers but instead accumulate directly into oligomers and higher-order aggregates. In addition to larger aggregates, fragments with sizes around 1 S were detected with increasing TCEP incubation time. This fragmentation behavior was consistent among FLCs originating from the immunoglobulin kappa variable 1-33 gene (IGKV1-33). Sedimentation velocity-based characterization of FLCs can provide insights into the relationship between their stability and aggregation capacity. An understanding of this relationship is crucial for the development of therapeutic strategies to prevent renal complications associated with monoclonal gammopathies such as multiple myeloma.

**Supplementary Information:**

The online version contains supplementary material available at 10.1007/s00249-025-01788-2.

## Introduction

Immunoglobulins form the humoral component of the adaptive immune system and are secreted by plasma cells in the bone marrow after their terminal differentiation from B lymphocytes. They consist of paired heavy and light polypeptide chains that are covalently linked via disulfide bonds, resulting in a Y-shaped structure (Fleischman et al. 1963; Edelman et al. 1968; Amzel and Poljak 1979). When light chains are released into the serum unbound to their heavy chain counterpart, they are referred to as free light chains (FLCs). Excessive production of monoclonal protein is a key pathological feature of a heterogeneous group of diseases known as monoclonal gammopathies (Ríos-Tamayo et al. 2023). As a result of malignant transformation, clonal expansion of a single neoplastic plasma cell can lead to the continuous overproduction of monoclonal FLCs. Under these pathological conditions, elevated FLC levels can be detected in the serum, cerebrospinal and synovial fluid and urine, where they are also referred to as Bence Jones protein (Jones 1848; Kyle 2000). In light chain amyloidosis (AL amyloidosis), these elevated levels of circulating FLCs lead to the formation and deposition of highly ordered amyloid fibrils, which progressively disrupt the function of multiple organ systems (Kaplan et al. 2014; Merlini et al. 2018). In multiple myeloma (MM), the clinical focus often centers on renal damage caused by the accumulation of insoluble FLC deposits in the renal tubules (Sathick et al. 2019). These deposits also manifest as geometrically shaped crystalline casts or amorphous and granular aggregates often lacking a defined high-order structure (Zanetti and Capra 1999; Bliznyukov et al. 2005; Sun et al. 2021; Sternke-Hoffmann et al. 2023). Each form is distinguishable by its morphology and clinical relevance and the microenvironment has been shown to play a critical role in determining whether and how those different types of aggregates form (Bliznyukov et al. 2005).

Due to the structural diversity of FLCs and the variety of clinical manifestations, the underlying aggregation mechanism remains largely unclear. It is assumed that the severity of this damage is often linked to the structural properties of FLCs and their ability to interact with different tissue types (Del Pozo-Yauner et al. 2022; Gudowska-Sawczuk and Mroczko 2023). Structurally, light chains are divided into the isotypes Kappa (*κ*) and Lambda (*λ*), each consisting of a constant (*C*_L_) and a variable (*V*_L_) domain, adopting a *β*-sandwich structure. A set of different gene segments encodes the *C*_L_ and *V*_L_ domains of both *κ*- and *λ*-light chains. The immunoglobulin kappa variable 1-33 (IGKV1-33) gene segment is the most frequently expressed variable *κ*-domain gene in AL amyloidosis and is associated with both higher dFLC levels—defined as the difference between involved and uninvolved FLC concentrations—and an increased incidence of hepatic involvement (Kourelis et al. 2017). Three of the nine FLC samples analysed in this study (P006, P016, and P017) originated from IGKV1-33 (Sternke-Hoffmann et al. 2023). Light chains encoded by this gene exhibit a greater number of somatic mutations (Morgan et al. 2024), a feature that may underlie the distinct fragmentation behavior reported here. Both isotypes contain around 214 amino acids each with a molecular weight of approximately 25 kDa. They typically include five cysteine residues forming disulfide bonds; two intramolecular bonds stabilizing the monomeric conformation, while a third C-terminal cysteine is associated with covalent dimerization (Janeway 2001; Thio et al. 2008; Liu and May 2012). While *κ*-FLCs are mostly present as monomers, *λ*-FLCs form dimers, with dimerization generally occurring through both covalent and non-covalent interactions (Klein et al. 1977; Roussel et al. 1999). Although dimers are typically kinetically stable and resistant to endoproteolysis, unstable forms have been identified that unfold more rapidly, increasing their susceptibility to proteolytic digestion and resulting in fragmentation (Morgan and Kelly 2016; Morgan et al. 2019). In particular, fragments of the N-terminal variable domain have demonstrated a high aggregation potential and have been identified as the primary component of amyloid fibrils (Glenner et al. 1970). The mechanisms regulating the formation of disulfide-bonded dimers remain largely unknown, but it is believed that they are catalyzed by oxidoreductases, whose activity depends on the redox potential of the cell compartment (Migrino et al. 2010). This redox potential can be altered by pathological conditions that include oxidative stress, thereby affecting the dithiol-disulfide state of FLCs. In various diseases, an abnormal increase in FLC dimerization has been observed, suggesting its pathological relevance (Kaplan et al. 2011). It is known that the disruption of disulfide bonds can trigger the aggregation of FLCs (Andrich et al. 2017). A variety of reducing agents can be utilized for this purpose in vitro, and studies have demonstrated that the choice of reducing agent significantly impacts both the kinetics of aggregation and the morphology of the resulting aggregates (Džupponová and Žoldák 2023).

This study investigates the impact of reducing conditions on the molecular species of FLCs during the aggregation process, aiming to explore a potential correlation between their propensity to aggregate and the extend of tissue damage. Therefore, nine pathological FLC samples purified from the urine of patients with multiple myeloma were characterized using analytical ultracentrifugation (AUC) (Krayukhina and Uchiyama 2016). Size and shape distributions of the FLC samples were determined under different conditions by sedimentation velocity (SV) analysis. Measurements were repeated after different incubation periods in the presence of the reducing agent tris(2-carboxyethyl)phosphine (TCEP) to investigate the role of intra- and intermolecular disulfide bonds in stabilizing FLCs and mitigating their aggregation. As an absolute technique based on fundamental physical principles, SV centrifugation provides a direct measurement without the need for reference standards or calibration (Schuck et al. 2016). A defined sample volume is subjected to high-speed centrifugation, which facilitates the separation of species based on their hydrodynamic properties and the determination of their respective sedimentation coefficients (*s*-values). This method allows the quantification of monomeric and dimeric FLC fractions, while also allowing the identification of higher order oligomeric species over a range of experimental conditions. In this study, we distinguish between physiologically occurring oligomeric forms—including both reversible (non-covalent) and irreversible (covalent) interactions—and pathologically relevant aggregates. In this context, ‘aggregation’ refers to the formation of irreversible, misfolded assemblies that arise from structural destabilization and are implicated in the pathogenicity of FLCs in MM and AL amyloidosis. In search for prognostic markers capable of providing insights into the expected course of disease, this study builds upon prior research conducted using the same set of patient samples (Sternke-Hoffmann et al. 2020, 2023; Dupré et al. 2021). Understanding the molecular changes in sample composition during aggregation and amyloid fibril formation may lead to valuable insights into the nephrotoxicity of FLCs.

## Methods

### Sample collection and purification

All samples analyzed in this work were obtained from a 24-h urine collection of both inpatients and outpatients at the University Hospital Düsseldorf. Prior to sample collection, all patients provided written informed consent, and the study was approved by the ethics committee of the University Hospital Düsseldorf (study number 5926R and registration ID 201706). Patient samples were prepared as described previously (Sternke-Hoffmann et al. 2020). Briefly, FLCs were isolated from collected urine of patients with multiple myeloma through ammonium sulphate precipitation. The precipitation reaction involved the saturation of collected urine with 70% (w/v) ammonium sulphate following incubation at 4 °C for 2 h with stirring on the day of collection. The solution containing precipitated protein fractions was then centrifuged at 6000×*g* and 4 °C for 25 min. To remove residual ammonium sulphate and other impurities, precipitate resuspended in 30 mM Tris–HCl buffer at pH 7.4 was dialysed against the same buffer for 72 h at 4 °C with gentle stirring. To maintain the concentration gradient, a buffer change was carried out every 24 h. The samples were fractionated using size-exclusion chromatography (SEC) with an ÄKTA pure chromatography system from GE Healthcare (Illinois, USA) and a Superdex 75 10/300 GL Tricorn column with a separation range of 3–70 kDa. Elution was performed using 30 mM Tris–HCl buffer at pH 7.4. Prior to SEC, samples were concentrated using Sartorius Vivaspin 2 centrifugal concentrators with polyethersulfone membrane and 10 kDa molecular weight cut-off (MWCO). Individual SEC fractions were measured in a V-650 spectrophotometer from Jasco (Pfungstadt, Germany). Based on the individual amino acid compositions of the FLC samples (Dupré et al. 2021), the respective extinction coefficient was calculated by the Edelhoch method (Edelhoch 1967) implemented in ProtParam (Expasy). Individual SEC fractions were combined according to their concentration, shock-frozen with liquid nitrogen and stored at −80 °C until further use.

### Quantification of species distributions with SV-AUC

SV experiments were performed in ProteomeLab XL-A and Optima XL-A ultracentrifuges from Beckman Coulter (California, USA) equipped with UV/Vis absorption optics. Standard double-sector measuring cells with quartz glass windows and aluminum centerpieces with 12 mm optical path length were used, along with an An-60Ti 4-hole rotor (Beckman Coulter). A reference volume of 400 µL and a sample volume of 390 µL were used for all measurements. Unless stated otherwise, all AUC experiments were performed with sample concentrations of 35 µM in 30 mM Tris–HCl buffer at pH 7.4. To promote disulfide bond reduction, the samples were incubated with 7 mM TCEP adjusted to pH 7.4 for 1.25 h, 5 h or 15 h at 37 °C with shaking at 600 rpm, prior to loading samples into the AUC cells. Alternatively, 0.1 M and 1 M NaCl were added to the samples following 24 h incubation at room temperature to evaluate how changes in ionic strength affect the species distribution of FLCs. As different samples were measured together per run, the wavelength for sample detection was individually set for optimal resolution (between 260 and 270 nm) based on wavelength scans taken at 3000 rpm before each measurement. The SV experiments were carried out at a rotor speed of 60,000 rpm and a temperature of 20 °C. After loading, samples were equilibrated in the rotor at 20 °C for approximately 60 min prior to the start of sedimentation. This equilibration period included vacuum establishment, temperature stabilization, and low-speed wavelength scans at 3000 rpm, ensuring thermal and hydrodynamic equilibrium before high-speed centrifugation. SV experiments were carried out at the maximum rotor speed of 60,000 rpm, which was sufficient to achieve complete sedimentation of monomeric, dimeric, higher-order oligomeric species, and aggregates within approximately 5 h. Theoretical estimates indicate that monomers sediment in about 4.5 h and dimers in approximately 3.5 h under these conditions. Low molecular weight fragments were not considered here, as their sedimentation is minimal over the course of the experiment.

### Calculations and data analysis

For the analysis of sedimentation data, the partial specific volume, as well as solvent density and viscosity of all FLC samples were calculated individually using the software SEDNTERP Version 2.0 Beta (Laue 1992). Details of all fit parameters used for analysis are provided in the Supplementary Material (Table S1). The sedimentation data analysis was carried out with the software SEDFIT Version 16.1c using the continuous distribution *c*(*s*) Lamm equation model (*c*(*s*) model) (Schuck 2000; Schuck et al. 2002). A resolution of 0.1 S was applied for curve fitting, and the root mean square deviation (RMSD) was less than 1% of the total signal for all sedimentation data, except for sample P020 after 5 and 15 h of incubation with TCEP. Both time-invariant (TI) and radial-invariant (RI) noise parameters were fitted in SEDFIT. Trial fits with the RI parameter disabled produced identical low-S and high-S regions, confirming that RI-noise modelling did not introduce artifacts. Significant aggregation and sedimentation of aggregates during the acceleration of the rotor to the final speed of 60,000 rpm resulted in a notable loss of signal, thereby affecting the signal-to-noise ratio. For these measurements, the achieved RMSD values were approximately 2% of the total signal. Sample loss was calculated by comparing the absorption signal from wavelength scans at the respective detection wavelength before measurement with the plateau heights recorded in early sedimentation profiles for each sample. Despite the low ionic strength of the buffer (30 mM Tris–HCl, pH 7.4), no significant primary-charge effect was observed in the sedimentation behavior of the FLCs. The measured s-values for monomeric and dimeric FLCs closely match values reported in the literature under different buffer conditions, as well as values obtained in this study under higher ionic strength, supporting the robustness of our sedimentation analysis. For further analysis and graphical representation fitted sedimentation data were exported to the software GUSSI (Brautigam 2015) and DataGraph Version 5.3β, copyright 2020 (North Carolina, USA). The *c*(*s*) distributions obtained show the number and *s*-values of the molecular species modelled as constitutes of the analysed samples. The *s*-values obtained from *c*(*s*) analysis were corrected to standard conditions (20 °C in water) and are reported as *s*_20,*w*_ values. Default subtraction of TI and RI noise was applied in GUSSI. Integration limits including all detected peaks of the distribution were used to determine the weight-average *s*-values of a sample. This approach does not require baseline resolved signals of single species in the distribution and provides a mean value reflecting the overall oligomerization state of the FLC samples. Within these global integration limits, individual ranges for fragments, monomers, dimers, and tetramers were selected for each sample, as far as possible.

### Circular dichroism spectroscopy

The extent of secondary structure changes in FLCs upon TCEP treatment over various incubation times was determined by circular dichroism (CD) spectroscopy. FLC samples, prepared at an initial concentration of 35 µM in 10 mM sodium phosphate buffer at pH 7.4, were incubated with 7 mM TCEP at 37 °C. To minimize aggregation, samples were not agitated, which allowed a more controlled analysis of structural changes. Aliquots were taken at 1.25 h, 5 h and 24 h and subsequently diluted to a final FLC concentration of 9.23 µM for CD spectroscopy. Since the final concentration of each sample was calculated and achieved through dilution, potential variations due to aggregation cannot be ruled out. Therefore, ellipticity values were not normalized to molar ellipticity to account for any inconsistencies that might arise from such aggregation effects. Far-UV CD spectra were measured using a Jasco (Hachioji, Tokyo, Japan) J-810 spectropolarimeter at room temperature across the wavelength range of 190 nm to 260 nm. Measurements were performed in quartz glass cuvettes with 1 mm optical path length, a scanning speed of 50 nm/min, 1 nm bandwidth, a digital integration time of 2 s and a resolution of 0.5 nm. Each spectrum was baseline corrected and represents the average of 15 accumulations.

### Differential scanning fluorimetry

Differential scanning fluorimetry (DSF) can be used to assess the thermal stability of a sample across various conditions, enabling the detection of shifts in melting temperature that indicate structural unfolding. For analysis of the thermal stability of the FLCs a CFX Opus 96 Real-Time PCR System (Bio-Rad, California, USA) was used. All assays were performed at a sample concentration of 35 µM in a 30 mM Tris–HCl buffer at pH 7.4. To destabilize the disulfide bonds of FLCs, 7 mM TCEP was added to each preparation. For stability assessment at high ionic strength, the experiments were repeated with 0.1 M and 1 M NaCl. All measurements were performed in triplicate, with a total reaction volume of 25 µL per well. Real-time unfolding of FLCs was monitored using the SYPRO Orange fluorescent dye (Sigma-Aldrich, S5692). A 10 mM stock solution was diluted 1:1000 in the reaction mixture, resulting in a final working concentration of 10 µM. Samples were pipetted into low-profile, thin-walled Hard-Shell 96-well PCR plates (#HSP9601) and sealed with Microseal 'B' adhesive PCR plate sealing film (#MSB-1001). Melting curves were measured from 10 to 95 °C, with a temperature increment of 0.5 °C per cycle every 15 s.

## Results

### SV-analysis of the size distribution of FLC samples

**Fig. 1:**
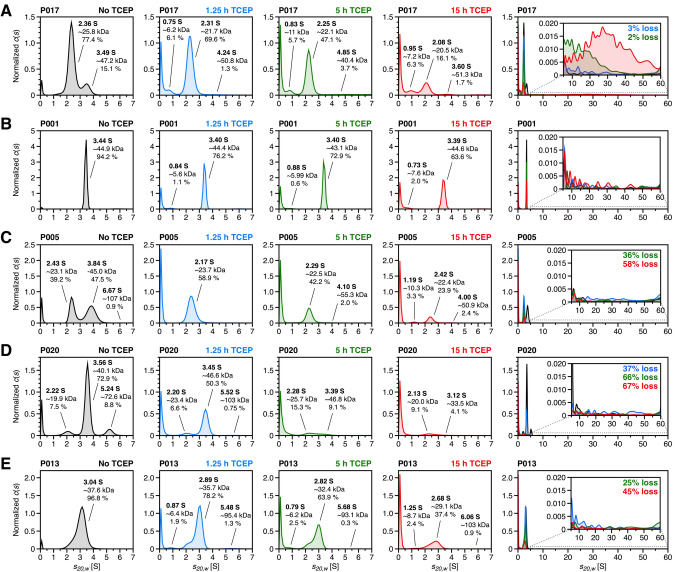
Influence of disulfide bond reduction on the monomer–dimer distribution of FLCs. The *c*(*s*) distributions of purified patient samples **A** P017, **B** P001, **C** P005, **D** P020 and **E** P013 are shown. Samples were prepared at a concentration of 35 µM in 30 mM Tris–HCl, pH 7.4 (black). To induce disulfide bond reduction, 7 mM pH adjusted TCEP was added, followed by incubation at 37 °C with shaking at 600 rpm for periods of 1.25 h (blue), 5 h (green), and 15 h (red). After incubation, 390 µL of each sample was transferred to the sample sector of standard aluminum double-sector cells, and sedimentation analysis was performed at 60,000 rpm and 20 °C

Purified FLC samples were subjected to SV analysis to determine their oligomeric state, expressed as *s*-value distribution. As described before, FLCs showed significant differences in size distributions under neutral buffer conditions (Sternke-Hoffmann et al. 2023). Briefly, determined *s*-values could be assigned predominantly to monomeric and dimeric FLC species in agreement with literature values, i.e. ~2.4 S for monomers and ~3.65 S for dimers (Klein et al. 1977). As also illustrated in Fig. 1 (distributions without TCEP), all FLC samples showed distinct species distributions, with *λ*-FLC P001 being particularly notable as the only sample with no detectable monomeric species, consisting almost entirely of dimers. Other samples showed either a higher proportion of monomers or dimers or, in the case of sample P005, an almost even distribution. In samples P007, P013, and to a lesser extent P016, the signals for monomers and dimers could not be distinctly resolved, resulting in one asymmetric peak with a left-skewed distribution and a variably pronounced shoulder. This suggests that the *s*-values of monomers and dimers in these samples are sufficiently close to hinder clear separation or that a dynamic, non-covalent equilibrium exists between species, preventing distinct detection over the several-hour measurement. In all distributions, additional signals of varying intensity were detected around ~0.1 S. These signals could potentially be artifacts resulting from the calculation of *c*(*s*) distributions using maximum entropy regularization (Schuck 2016). Alternatively, they may reflect the presence of non- or slowly sedimenting solvent components or proteolytic fragments present in the samples. Additionally, samples P004 and P020 exhibit signals around ~5 S, which may correspond to impurities such as HSA or potentially represent trimeric or tetrameric FLC species, as described in the literature (Sakai et al. 2023). While this signal was weakly pronounced in sample P004, it was most prominent in sample P020, where it accounted for 8.8% of the total sample.

### Changes in FLC size distribution after disulfide bond reduction

Building on previous SV experiments, 7 mM TCEP was added to the buffer (30 mM Tris–HCl, pH 7.4) to disrupt the disulfide bonds of FLCs. Given that this process reduces both intra- and intermolecular disulfide bonds, it is to be expected that both the tertiary and quaternary structures will be impaired. As demonstrated, these conditions are sufficient to destabilize the FLC structure to a degree that initiates self-assembly (Džupponová and Žoldák 2023). Since TCEP is acidic when dissolved in water, stock solutions were adjusted to pH 7.4 to maintain neutral pH conditions in samples after TCEP addition. The samples were incubated for 1.25 h, 5 h, and 15 h under reducing conditions at 37 °C with shaking at 600 rpm. The size-distribution analysis captures changes induced by the onset of aggregation, regardless of the specific type of aggregation involved. Figure 1 presents selected *c*(*s*) distributions, chosen based on their distinct species composition or individual response to the reducing conditions. The remaining distributions are included in the Supplementary Material (Fig. S1), along with the corresponding raw data (Fig. S2). By comparing the *c*(*s*) distributions across different incubation times, the changes in molecular species in FLCs can be tracked throughout the aggregation process. After 1.25 h of incubation, the dimeric species in samples P017 (Fig. 1A) and P005 (Fig. 1C) almost completely dissociate. In samples with a higher initial dimer content, such as P001 (Fig. 1B), P020 (Fig. 1D) and P013 (Fig. 1E), a continuous decrease in dimer levels is observed with increasing incubation time. In sample P020, dimers exhibit substantial dissociation after 15 h of incubation. Similarly, sample P013 shows a shift in the *s*-values towards monomeric species with increasing incubation time, indicating that dimeric components progressively dissociate over time. Notably, only sample P001 retains a significant proportion of dimers after 15 h of incubation. This observation suggests that the remaining dimeric species in sample P001 are likely not covalently linked but instead stabilized by non-covalent interactions, which remain unaffected by the reduction of disulfide bonds.

Over time, the proportion of monomeric species also decreases, accompanied by a decline in *s*-value by an average of 0.2 S. This change could indicate structural changes, possibly due to the unfolding of monomers after the reduction of intramolecular disulfide bonds, which could explain the observed variations in sedimentation behavior. Unexpectedly, no increase in monomeric species was observed following the dissociation of dimers, particularly noticeable in sample P001. The exception was sample P005, where the proportion of monomers increased from ~39 to 59% after an incubation time of 1.25 h. These findings suggest that most monomers dissociating from reduced dimers exhibit distinct stability characteristics compared to free monomers, directing them immediately into the aggregation pathway. In addition to the redistribution of monomeric and dimeric species, an increase in signal at approximately 0.1 S is observed with prolonged incubation times. To confirm that the low-S signals represent genuine sample components rather than artifacts of maximum-entropy regularization, sedimentation data was re-analyzed with several minimum sedimentation-coefficient limits (*s*_min_) both with and without Bayesian modification to suppress baseline correlation. As shown in Supplementary Material (Fig. S3), half-peaks appear only when *s*_min_ exceeds 0 S, whereas setting *s*_min_ to 0 S and applying Bayesian modification recovers the complete peak at ~0.1 S, indicating that these low molecular weight species are intrinsic to the samples and not mathematical artifacts (Schuck 2016). Since all measurements were performed under identical buffer conditions, the observed increase in signal cannot be attributed to solvent components. Instead, it may be associated with the degradation of monomeric FLCs, as indicated by their decreasing *s*-values, resulting in the formation of low molecular weight fragments. Additionally, the formation of a new species with an *s*-value of approximately 1 S was observed in samples P017 (Fig. 1A), P006 and P016 (Fig. S1), and to a lesser extend in sample P005 (Fig. 1C), with increasing TCEP incubation time. In contrast to the previously identified low molecular weight fragments, this signal may indicate an alternative degradation mechanism occurring at a specific site within the protein sequence of FLCs.

**Fig. 2:**
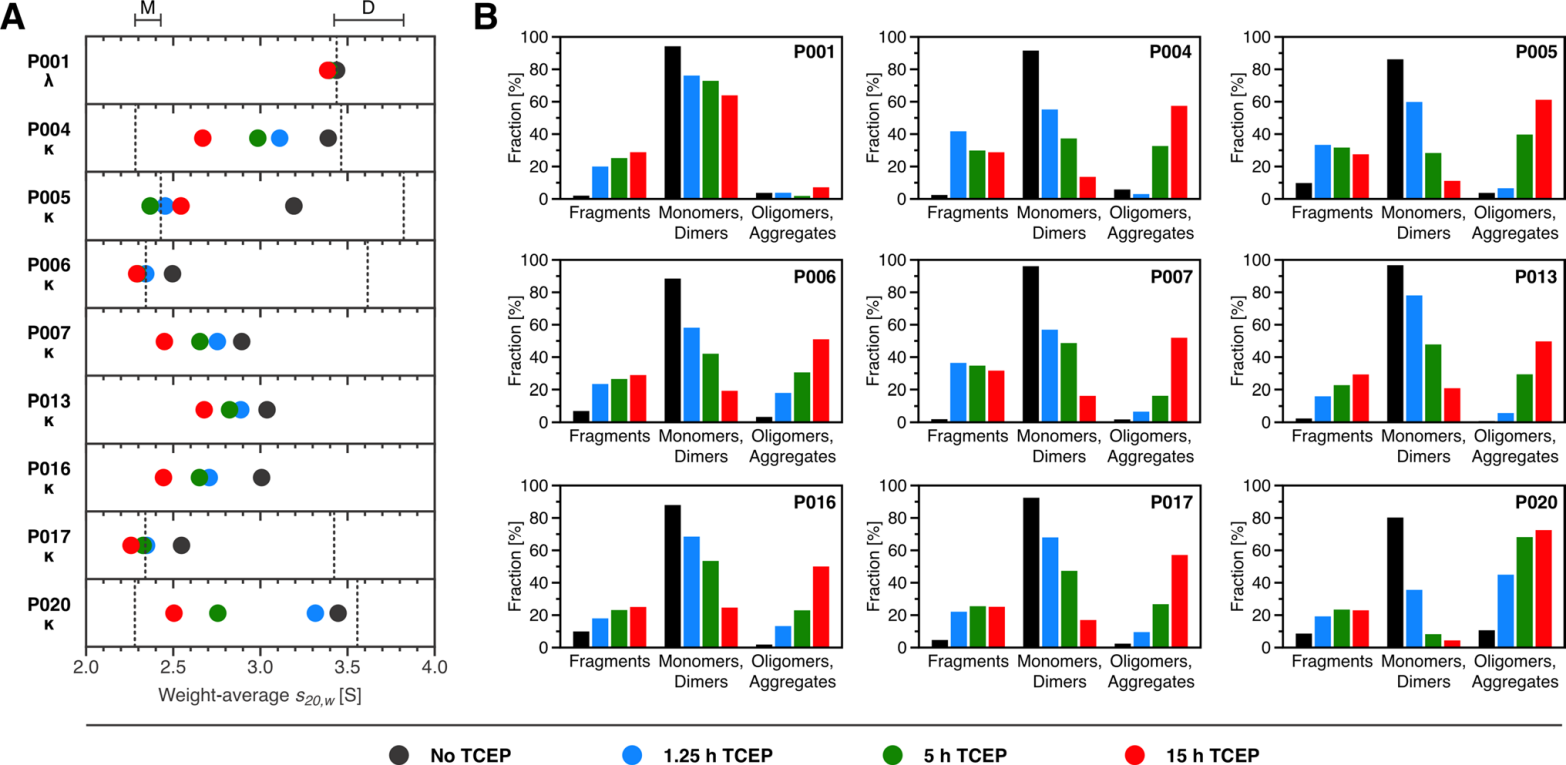
Shifts in monomeric and dimeric weight-average *s*-values and in time-dependent size distributions during TCEP incubation. **A** Illustration of the changes in weight-average *s*-values for all patient samples, derived from the analysis of sedimentation data. To track changes in monomeric and dimeric species over the incubation period, the sedimentation range from 1.6 to 4.7 S was integrated to calculate the weight-average *s*-values for both species combined. Dashed lines indicate *s*-values for individually identified monomers and dimers, with 'M' and 'D' above representing their average *s*-value range across samples. **B** Percentage of all species over the entire *s*-value range. A distinction was made between fragments (0 to 1.6 S), monomers and dimers (1.6 to 4.7 S) as well as oligomers and aggregates (4.7 S to end of distributions). In addition to the proportions of aggregates determined by integration, the previously calculated loss of sample during sedimentation was also incorporated into analysis

**Fig. 3:**
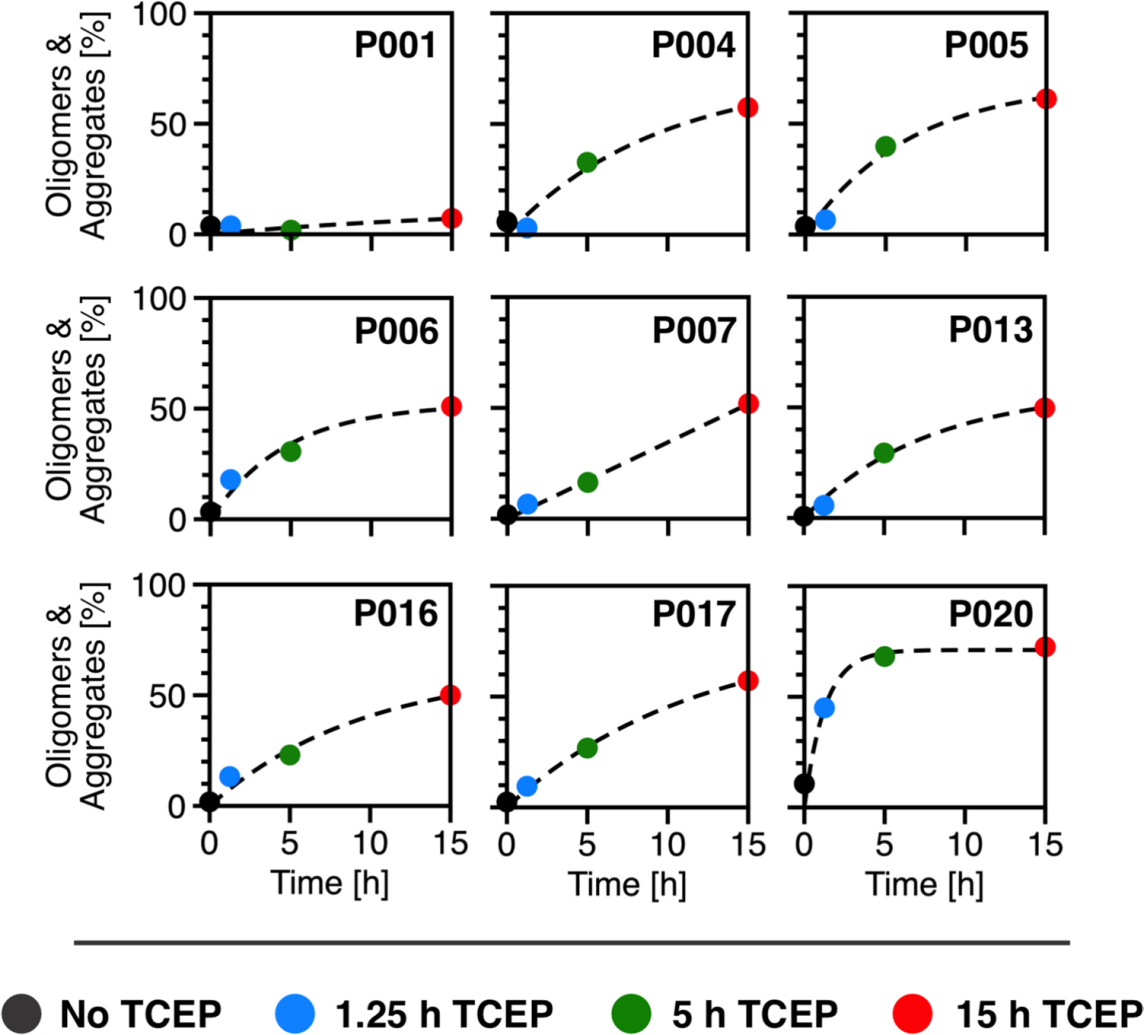
Time-dependent progression of TCEP-induced aggregation. Signal percentage of oligomers and larger aggregates (4.7 S–60 S) out of the total signal, calculated from the *c*(*s*) distributions for each patient sample. The progression of TCEP-induced aggregation is shown as a function of incubation time. Data points represent aggregate content of the samples without TCEP (black), and after 1.25 h (blue), 5 h (green) and 15 h (red) of incubation. An exponential growth function (*Y* = *A*(1–*e*^–*kx*^) was applied to illustrate differences in aggregation trends among samples over incubation time

The redistribution of molecular species in all samples triggered by the reduction of disulfide bonds is shown in Fig. 2B. By integrating respective single peaks of the *c*(*s*) distribution, the mass fractions of the corresponding molecular species present can be determined. For this, identical integration ranges were applied to all samples: 0 S to 1.6 S for fragments, 1.6 S to 4.7 S for monomers and dimers, and 4.7 S till the end of distribution range for oligomers and aggregates. The time-dependent shifts in the weight-average *s*-values of monomers and dimers during TCEP incubation are summarized in Fig. 2A. To provide a unified representation of the changes in sample compositions, monomeric and dimeric species were evaluated with common integration boundaries between 1.6 and 4.7 S. Additionally, Fig. 3 visualizes the time-dependent increase in TCEP-induced aggregation of the FLCs over the course of experiments, based on the calculated fractions.

**Fig. 4:**
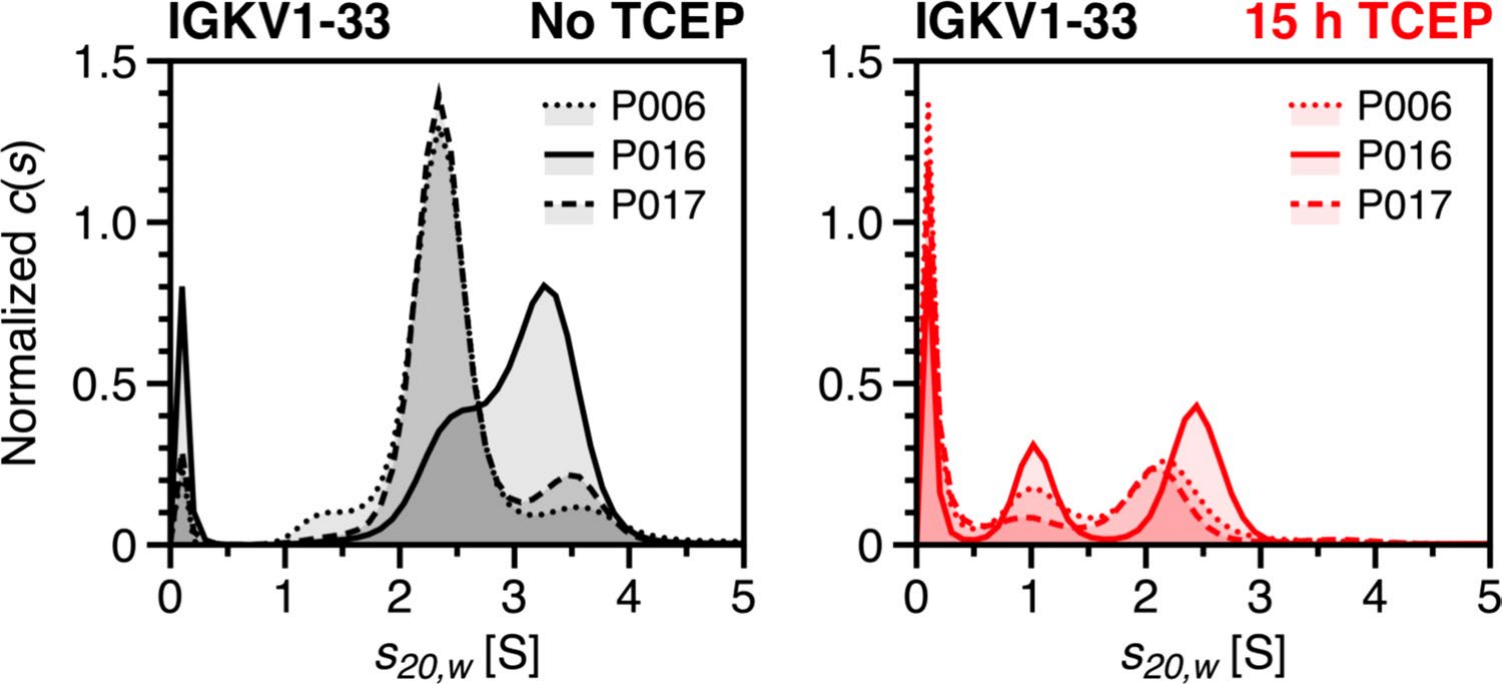
Fragmentation of IGKV1-33-derived FLCs after incubation with TCEP. Fragmentation patterns of FLCs derived from the IGKV1-33 germline sequence. The *c*(*s*) distributions of samples P006 (dotted line), P016 (solid line) and P017 (dashed line) are shown before TCEP addition (black) and after 15 h incubation with TCEP (red). To provide a more detailed comparison, only the range between 1 and 5 S is displayed

Incubation with TCEP revealed that patient samples P006, P016 and P017 exhibited a pronounced fragmentation pattern that intensified with increased incubation time. Notably, all three patient samples originate from the IGKV1-33 germline sequence, as previously identified (Sternke-Hoffmann et al. 2023). Figure 4 shows all three IGKV1-33 FLCs in comparison, both without TCEP and after 15 h of TCEP incubation. Along with the non-specific low molecular weight fragments observed across all patient samples, a defined fragment appeared at approximately 1 S. Prior to TCEP treatment, the monomer peak of sample P006 shows a left shoulder between 1 and 2 S, suggesting a potential fragmentation state after purification. However, this fragment does not remain consistent throughout the incubation series, likely undergoing further degradation until stabilizing as a fragment around 1 S, similar to those observed in samples P016 and P017. Fragmentation of FLCs may have been induced by a range of factors. Current knowledge suggests that kinetically unstable and partially unfolded dimers exhibit a higher susceptibility to proteolytic digestion, leading to the cleavage of FLC domains into individual fragments that may possess amyloidogenic properties (Glenner et al. 1970; Morgan and Kelly 2016). As detected by mass spectrometry analysis of the nine samples, various cathepsins were co-purified during FLC extraction from patients urine (Dupré et al. 2021; Sternke-Hoffmann et al. 2023). However, without the addition of TCEP, the FLC samples appeared to be stable over time, consistent with previous studies using the same set of FLCs (Sternke-Hoffmann et al. 2020, 2023). Given that the fragments at ~1 S are undetectable under non-reducing conditions, it can be assumed that the corresponding cleavage sites are exposed by the addition of TCEP. Furthermore, TCEP-induced fragmentation has been observed in other proteins, where TCEP facilitated nonspecific cleavage near cysteine residues over longer incubation times (Liu et al. 2010). Whether this fragmentation behavior is caused by TCEP or arises from proteolytic activity, and whether it represents a characteristic feature across all IGKV1-33-derived light chains requires further investigation.

### TCEP-induced changes in secondary structure of FLCs

**Fig. 5:**
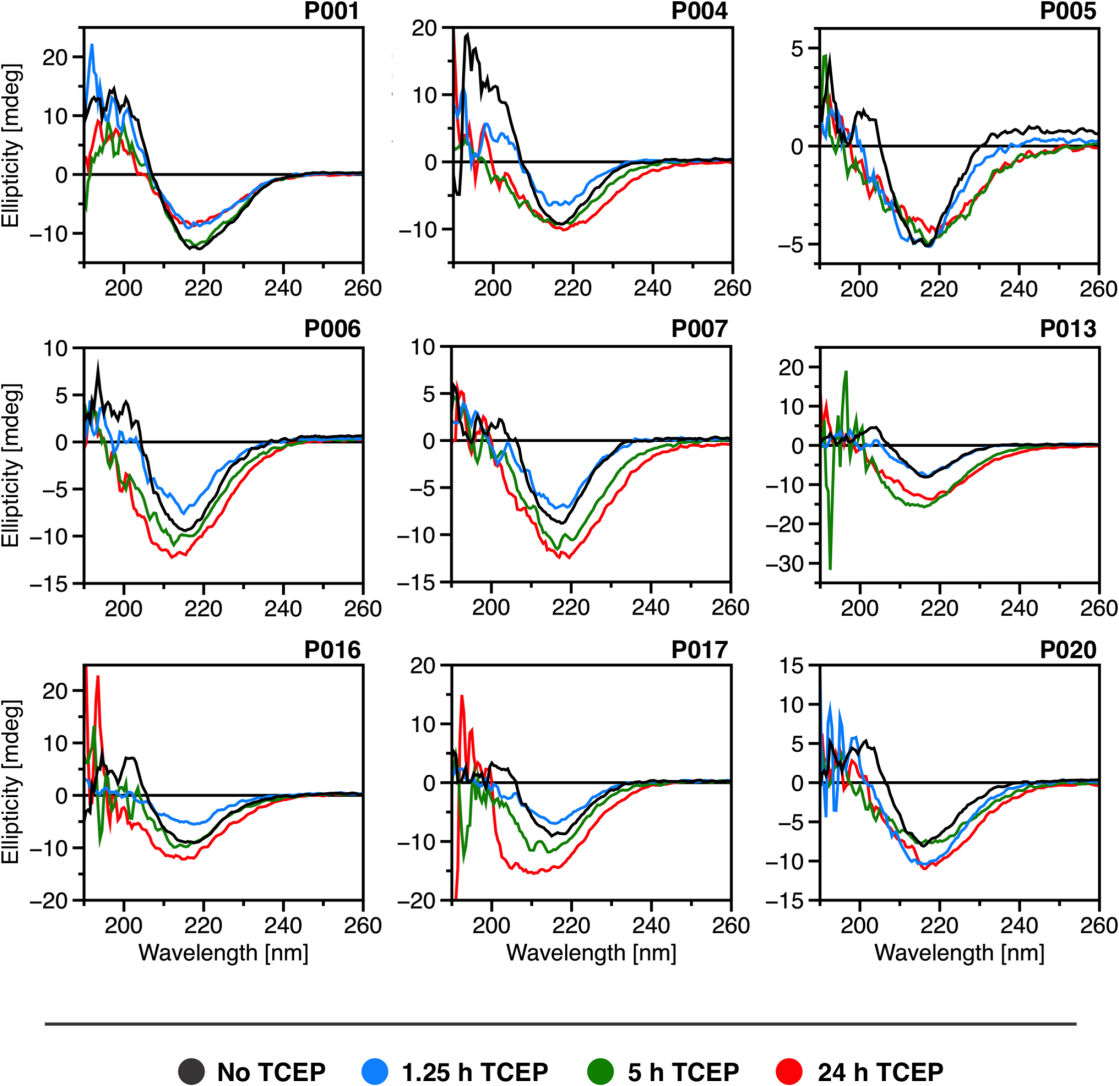
Secondary structure shifts after TCEP-induced reduction of disulfide bonds. Shown are the CD spectra of all nine FLCs before TCEP treatment (black), as well as after 1.25 h (blue), 5 h (green) and 24 h (red) incubation time. A sample concentration of 35 µM was incubated in 10 mM sodium phosphate buffer, pH 7.4 at 37 °C. Aliquots were taken after specified time points and diluted to a final concentration of 9.23 µM for CD measurements. Corresponding HT voltage records are provided in the Supplementary Material (Fig. S4)

To investigate possible changes within the secondary structure of FLCs caused by disulfide bond reduction through TCEP treatment, FLC samples were analyzed by CD spectroscopy. Figure 5 presents CD spectra for the nine FLC samples, both untreated and following incubation with TCEP at intervals of 1.25 h (blue), 5 h (green) and 24 h (red). In general, all FLC samples exhibit distinct β-sheet secondary structure motifs characteristic of light chains and other immunoglobulin domains (Brahms et al. 1977; Bork et al. 1994; Poshusta et al. 2013; Andrich et al. 2017). To monitor TCEP-induced changes across incubation periods, the zero-crossing of ellipticity in the range of ~200–210 nm was selected as a reference point. This point lies between the characteristic positive (~195 nm) and negative (~218 nm) bands typically observed in β-sheet-rich proteins such as FLCs (Greenfield 2006), and consistently appeared across all samples and time points. While not a structure-specific feature, the zero-crossing reflects the spectral balance of overlapping secondary structure contributions. Importantly, it remained robust despite minor differences in protein concentration or aggregation, making it a reliable and structurally meaningful marker for comparative analysis. Each sample exhibited varying levels of structural deviation from their initial structure after TCEP-induced disulfide bond reduction. Samples P001, P004 and P006 demonstrated minimal shifts at the zero-crossing point after 1.25 h incubation time, indicating an isosbestic point and limited structural disruption. In contrast, samples P005, P007, P017 and P020 displayed deviations from their initial structure, with zero-crossing shifts reaching up to 5 nm within the first 1.25 h of incubation, most notably in samples P005 and P020. These two samples also exhibited the highest aggregation levels after 15 h of incubation (Fig. 2B), suggesting a correlation between early structural loss and an increased aggregation propensity. Aside from sample P017 after 15 h incubation, post-incubation spectra across samples were generally similar in shape, with changes primarily attributable to aggregate formation and corresponding variations in effective sample concentration. Quantitative deconvolution was not attempted because reliable interpretation requires a high tension (HT) voltage below 700 V for wavelengths under 200 nm (Fig. S4), as well as a homogeneous, non-scattering sample. These criteria were not met by several spectra in this heterogeneous sample set. The zero crossings determined for all spectra are summarized in the Supplementary Material (Table S2).

### Modulating effects of the molecular species distribution of FLC

**Fig. 6:**
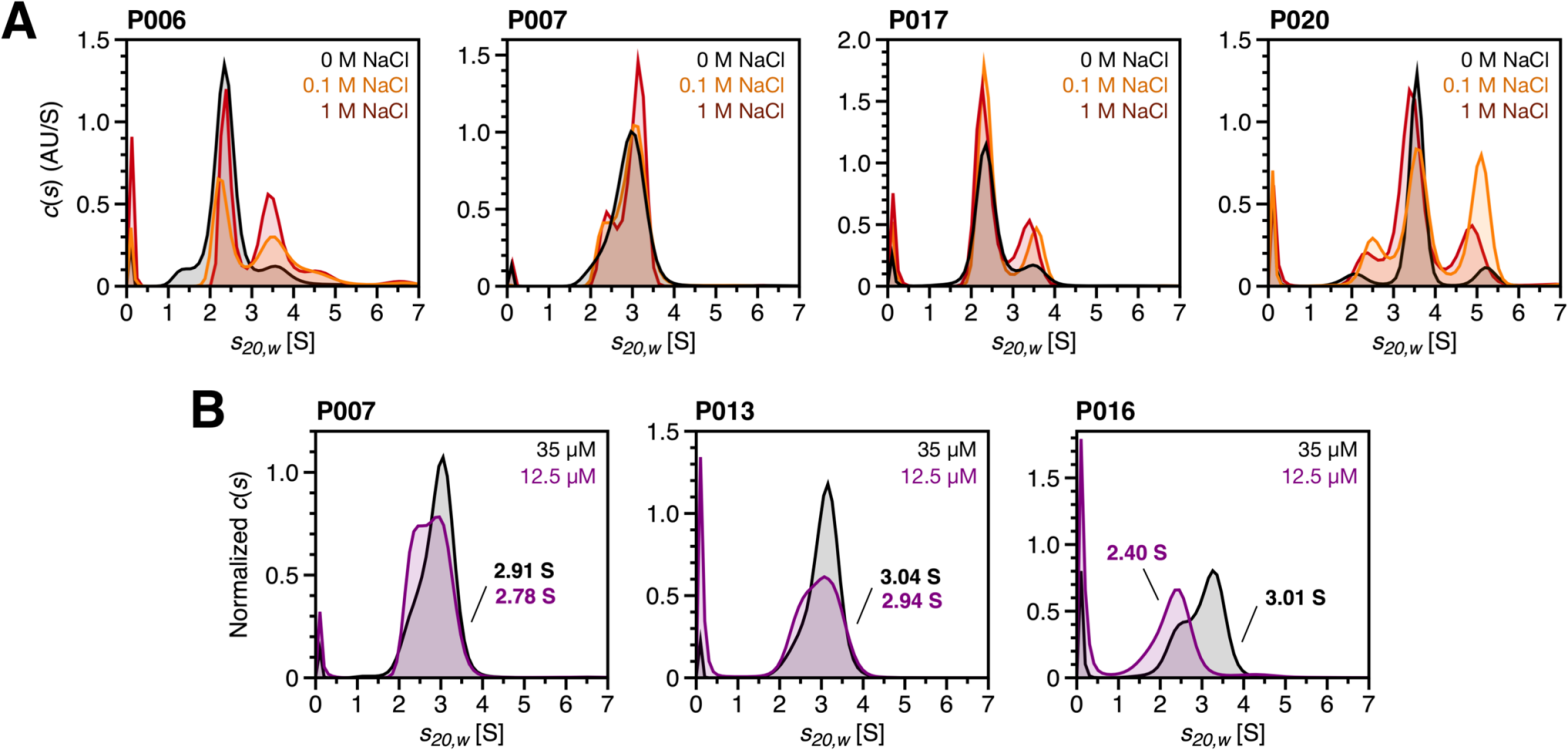
Modulation of the molecular species distribution of FLCs. **A** Effect of varying salt concentrations on the molecular species present in patient samples. The *c*(*s*) distributions of samples P006, P007, P017 and P020 are shown. A 30 mM Tris–HCl buffer at pH 7.4 was prepared without NaCl (0 M, black) and with the addition of 0.1 M (orange) or 1 M NaCl (red). To reach equilibrium, the samples were incubated for 24 h at 25 °C prior to measurement. **B** Concentration-dependent dynamic equilibrium between monomers and dimers. The *c*(*s*) distributions of samples P007, P013 and P016 at a concentration of 12.5 µM (purple) are compared with those obtained at 35 µM (black). Corresponding raw data of the *c*(*s*) distributions are included in the Supplementary Material (Figs. S7, S8)

Distinct salt concentrations across renal compartments are crucial for maintaining normal kidney function by enabling water and electrolyte homeostasis through active and passive transport mechanisms (Gallardo and Vio 2022). Ionic strength is known to significantly influence protein stability by modulating secondary and tertiary structures through both hydrophobic interactions (Von Hippel and Wong 1965; Damodaran and Kinsella 1981) and electrostatic forces, including charge repulsion and ion pairing, which are key determinants of folding energetics (Dill 1990). As a result, the dynamic osmotic gradient in the kidney may play a key role in the formation of nephrotoxic FLC aggregates. Previous studies have shown that varying salt concentrations can either promote or inhibit aggregate formation, depending on the specific FLC involved (Baden et al. 2009). To evaluate the relationship between ionic strength and FLC stability, patient samples were incubated with 0.1 M and 1 M sodium chloride (NaCl), and SV experiments were repeated under these conditions. Figure 6A displays the *c*(*s*) distributions of samples P006, P007, P017 and P020, illustrating the effects of ionic strength on FLC species distribution. Individual *c*(*s*) distributions with additional labeling are provided in the Supplementary Material (Fig. S5), along with a summary plot illustrating changes in weight-average *s*-values as a function of ionic strength (Fig. S6). For samples P006, P017 and P020, an increase in ionic strength correlates with a higher degree of oligomerization. While this observation applies only to the proportion of dimers in sample P017, the addition of NaCl also results in an increase in tetrameric species around 5 S in samples P006 and P020, with the effect being particularly pronounced in P020. Based on the observed signal increase, it can be assumed that the signal at ~5 S in sample P020 is unlikely linked to an HSA impurity, as previously hypothesized, but is more likely attributable to tetrameric FLC species. The most significant increase is observed at 0.1 M NaCl, whereas this signal decreases partly at 1 M NaCl. The addition of NaCl also results in sharper peaks, which is at least partly due to the reduced diffusion in the increasingly viscous salt solutions. For sample P007, the addition of NaCl facilitates the separation of the main signal, suggesting that a dynamic equilibrium between monomeric and dimeric species becomes kinetically stabilized. The increased ionic strength likely slows the interconversion between these states on the timescale of the experiment, allowing for clearer resolution of distinct species in the *c*(*s*) distribution. Nevertheless, the *s*-values for monomers and dimers remain relatively close to each other, preventing complete signal separation.

Depending on the type of FLC dimerization, a similar effect can be achieved by decreasing the sample concentration (Fig. 6B). At a concentration of 35 µM, samples P007, P013 and P016 showed no distinct separation between monomeric and dimeric signals (Figs. 1E, S1C, D). However, measurements repeated at FLC concentrations of 12.5 µM demonstrated varying degrees of dynamic equilibrium between monomers and dimers, further supporting the presence of non-covalent dimerization. While for samples P007 and P013 the peak shoulder of the monomer became more defined, sample P016 showed an almost complete shift to monomeric *s*-values. This is also reflected in the respective *s*-value, which decreased by ~0.1 S for samples P007 and P013 and by ~0.6 S for sample P016 at a concentration of 12.5 µM. Notably, after dilution, sample P016 displays a single dominant peak at 2.40 S, which corresponds to the *s*-value range of FLC monomers. This supports the hypothesis that samples P007, P013 and P016 exhibit, at least partially, a dynamic monomer–dimer equilibrium indicative of non-covalent dimerization. The results suggest that at a concentration of 35 µM, signals within the distribution of samples P007, P013 and P016 likely do not correspond to distinct FLC species. Instead, these signals reflect a reaction boundary formed by the rapidly reversible interaction between monomers and dimers during sedimentation, resulting in an averaged mixed state of FLC monomers and dimers (Schuck 2010). Nonetheless, the reduction of intramolecular disulfide bonds during the TCEP experiment appears to contribute to the dissociation of non-covalently bound dimers, as indicated by the observed continuous decline in dimer content with increasing incubation time as shown in Figs. 1E and S1C, D.

### Thermal stability of FLCs under reducing conditions

**Fig. 7:**
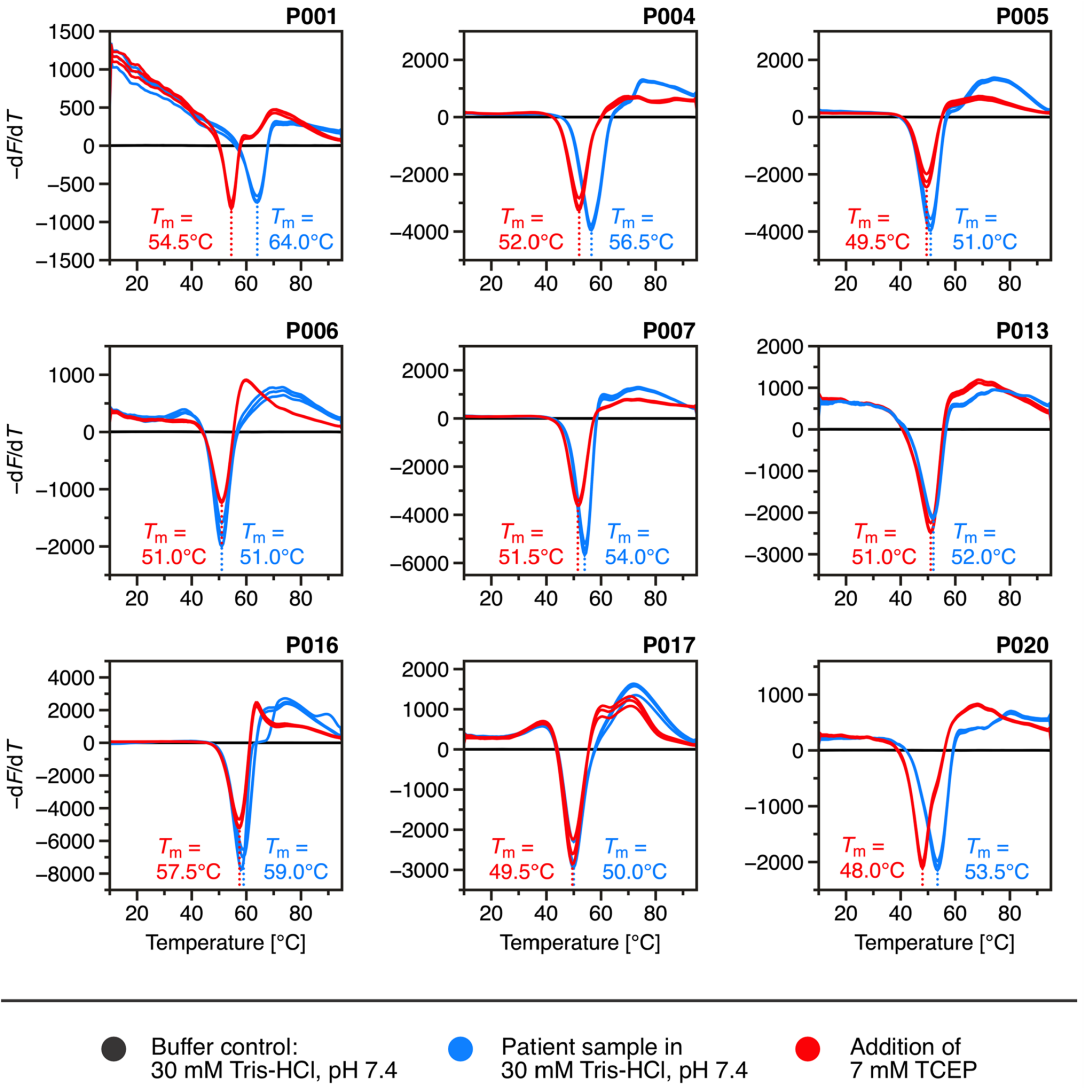
Influence of intra- and intermolecular disulfide bonds on melting temperatures of FLCs. The negative derivative fluorescence-temperature plots (−d*F/*d*T*) for all nine patient samples are shown. Samples were prepared at a concentration of 35 µM in 30 mM Tris–HCl, pH 7.4 (blue), with corresponding buffer control (black). To induce disulfide bond destabilization, 7 mM pH adjusted TCEP (red) was added. Thermal denaturation was monitored using 10 µM SYPRO Orange in a total volume of 25 µL per well, measured in triplicate. All triplicates are shown individually, and consistent curve shapes and *T*_m_ values were observed across replicates

To evaluate the stability of FLCs under reducing conditions, thermal shift assays were conducted utilizing DSF (Fig. 7). This technique allows the detection of changes in stability by monitoring shifts in the melting temperature (*T*_m_) of proteins. The melting temperatures of the FLC samples were initially determined under neutral buffer conditions with 30 mM Tris–HCl, pH 7.4. The measurement was then repeated with the addition of 7 mM pH adjusted TCEP to destabilize intra- and intermolecular disulfide bonds. The influence of TCEP-induced aggregation was considered negligible in the DSF measurements, as the samples were not pre-incubated, and the thermal scans began immediately after TCEP addition. This minimized the time available for aggregation to occur before reaching the unfolding transition. This assumption is supported by our SV-AUC and CD results (Figs. 3, 5), which show that significant aggregation requires extended incubation under reducing conditions.

The results indicate that observed differences in melting temperature induced by the reduction of disulfide bonds correlate with the propensity of FLCs to form dimers and higher oligomers. As expected for a κ-FLC, sample P006 shows the lowest degree of oligomerization, with 78.3% monomer and 9.7% dimer. After the addition of TCEP, no change was observed in the previously determined melting temperature of 51 °C. In contrast, sample P004, consisting of 6.3% monomers and 84.7% dimers, showed a melting temperature shift of 4.5 °C. Similarly, sample P020, consisting of 7.5% monomers, 72.9% dimers, and 8.8% tetramers, exhibited a 5.5 °C difference between its two determined melting temperatures. Notably, both samples show an unusual high degree of dimerization for *κ*-type FLCs, which may already indicate a pathological deviation (Kaplan et al. 2011). As typical for *λ*-FLCs, sample P001 predominantly exists as a dimer (94.2%) and show no detectable monomeric signal. A temperature difference of 9.5 °C was observed for P001, representing the largest deviation among the patient samples before and after disulfide bond reduction. This could be explained by the fact that, while *κ*-FLC dimerization often involves non-covalent interactions, *λ*-FLCs favors dimerization over covalent disulfide bonds (Sölling 1976). Although baseline separation of monomers and dimers was not achieved for samples P007, P013, and P016, their *c*(*s*) distributions nevertheless reveal dimeric species, as illustrated by their weight-average *s*-values (Fig. 2A). These findings suggest a dynamic monomer–dimer equilibrium driven by non-covalent interactions. Thermal shift data (Fig. 7) further support this interpretation: despite measurable dimer fractions, only minimal *ΔT*_m_ values were observed in samples P006 (0 °C), P017 (0.5 °C), P013 (1 °C), as well as P005 and P016 (1.5 °C). Such minor shifts may indicate that these dimers are stabilized primarily by non-covalent interactions rather than by covalent bonds. Notably, sample P016 retains a relatively high melting temperature of 57.5 °C after TCEP treatment, implying that some structural stabilization mechanisms remain active despite the reducing conditions. Since non-covalent dimerization would likely persist in the presence of TCEP, it is conceivable that this form of dimerization contributes to the thermal stability observed in P016. Alternatively, sequence-specific features or mutations unique to P016 may account for its above-average thermostability, even in the reduced state. In some measurements, particularly in sample P001, a distinct pre-transition fluorescence signal was observed. As no precipitation or signal loss was detected by SV-AUC, this signal was attributed to interactions with the fluorescent dye with native light chains, partially unfolded species, or other components such as light chain fragments. Notably, this pattern was not consistent across all FLC samples and triplicates confirmed a high degree of reproducibility.

**Fig. 8:**
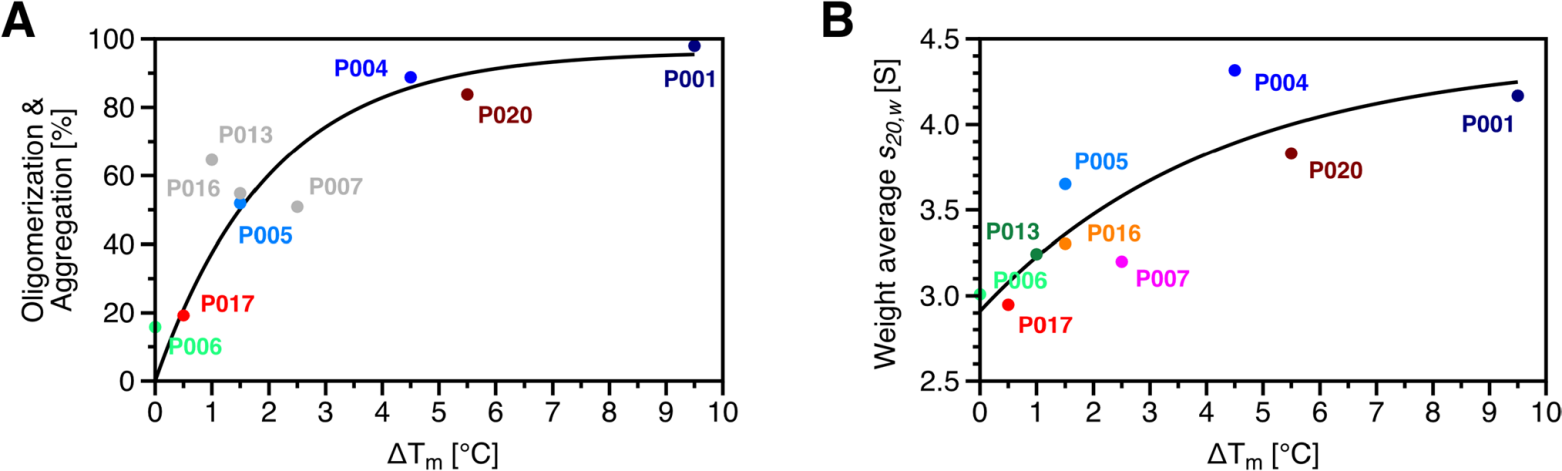
Correlation between oligomerization and the TCEP-induced melting-temperature shift (*ΔT*_m_). **A** Oligomerization as a function of Δ*T*_m_, obtained by integrating each *c*(*s*) distribution from 2.93 to 60.976 S, representing all species larger than monomer and low molecular weight fragments. The calculation is independent of that in Fig. 2, although both analyses draw on the same dataset. Due to monomer and dimer peak overlap in samples P007, P013 and P016, the same limits were applied for consistency. These data points (grey) were excluded from the fit analysis. **B** Relationship between *ΔT*_m_ and weight-average *s*-values calculated by integration of the full *c*(*s*) distribution range from 0 to 60.976 S, including all detected species, such as fragments, monomers, and oligomers. DSF measurements were conducted without pre-incubation following TCEP addition, to isolate the effect of inter- and intramolecular disulfide bond reduction on thermal stability. Because DSF scans began immediately after TCEP addition and lasted only ~1 h, additional aggregation or fragmentation during the measurement is negligible. Fragment peaks in the *c*(*s*) distribution profiles therefore reflect the samples’ initial heterogeneity, not TCEP-induced degradation. Consequently, the full *c*(*s*) range was used for calculation of weight-average *s*-values to capture the complete molecular composition and its relation to thermal stability

Figure 8A presents the relationship between melting temperature shifts and oligomerization, demonstrating the correlation between both variables. As oligomerization increases across FLC samples, shifts in melting temperature before and after TCEP treatment become more pronounced, reaching a maximum shift of approximately 10 °C for sample P001, which consists entirely of dimers. In Fig. 8B, shifts in melting temperature are presented as a function of the weight-average *s*-values of FLCs. This value, averaged over the total distribution, provides an additional measure of the oligomerization states, allowing an inclusion of samples P007, P013 and P016. It remains uncertain whether oligomerization beyond dimers would confer greater thermostability, with melting temperature shifts exceeding the highest detected shift of 10 °C. The findings demonstrate that oligomerization significantly enhances the thermostability of FLCs. Samples with a high monomer content display only minor shifts in melting temperature after TCEP treatment, further suggesting that the stabilizing effects of the intramolecular disulfide bonds are minimal. Instead, stability appears to be predominantly driven by covalent dimerization or oligomerization rather than solely through disulfide bonds.

The SV experiments indicate that sample P001 is unique among the samples in retaining dimeric structures after 15 h of incubation. This dimerization may result from non-covalent interactions, which could also influence the thermostability of the sample as assumed for samples P007, P013 and P016. Given that the lowest melting temperature measured after TCEP addition was 48 °C (in sample P020), it remains possible that the melting point of sample P001 could similarly decrease if complete loss of dimeric structure occurs. Consequently, the persistence of dimerization, even in reducing conditions, could play a critical role in maintaining the higher thermal stability observed in sample P001. Obtained melting temperatures and their respective shifts after disulfide bond reduction are summarized in Table 1. Corresponding thermal denaturation profiles for all patient samples are provided in the Supplementary Material (Fig. S9). In addition to measuring the melting temperatures under standard conditions, the impact of ionic strength on the thermal stability of FLCs was evaluated by adding 0.1 M and 1 M NaCl. DSF measurements were repeated under otherwise identical conditions. Thermal denaturation profiles and corresponding negative derivative fluorescence-temperature plots for all measurements are provided in the Supplementary Material (Figs. S10, S11). All melting temperatures, including those from the NaCl conditions, are compiled in Table 1. For most FLC samples, the addition of NaCl resulted in a concentration-dependent decrease in *T*_m_, both before and after TCEP treatment, suggesting that higher ionic strength may generally destabilizes these structures. However, this effect was not uniform across all samples. FLCs with more monomeric distributions, such as P006 and P017, showed minimal changes in *T*_m_, while more oligomeric samples, including P001, P004, and P020, exhibited more pronounced *T*_m_ reductions. This suggests that oligomeric FLCs may be more sensitive to changes in electrostatic interactions. Notably, samples derived from the IGKV1-33 germline again showed consistent behavior across different conditions.

**Table 1:** Melting temperatures of FLCs at different buffer conditions

				+0.1 M NaCl			+1 M NaCl		
Sample ID	*T*_m_ (°C)	*T*_m_^TCEP^ (°C)	*ΔT*_m_ (°C)	*T*_m_ (°C)	*T*_m_^TCEP^ (°C)	*ΔT*_m_ (°C)	*T*_m_ (°C)	*T*_m_^TCEP^ (°C)	*ΔT*_m_ (°C)
P001	64.0	54.5	9.5	61.0	53.0	8	61.0	51.5	9.5
P004	56.5	52.0	4.5	55.5	50.5	5	51.0	47.5	3.5
P005	51.0	49.5	1.5	49.5	48.0	1.5	47.5	46.5	1
P006	51.0	51.0	0	51.0	50.5	0.5	51.0	51.0	0
P007	54.0	51.5	2.5	53.0	50.5	2.5	51.5	50.0	1.5
P013	52.0	51.0	1.0	50.0	49.0	1.0	48.0	48.0	0
P016	59.0	57.5	1.5	59.0	57.0	2	60.0	59.0	1
P017	50.0	49.5	0.5	50.0	49.5	0.5	51.0	50.0	1
P020	53.5	48.0	5.5	51.5	46.5	5	51.0	45.5	5.5

*Δ* = *T*_m_ − *T*_m_^TCEP^

## Discussion

The biophysical characterization of FLCs purified from the urine of multiple myeloma patients aims to provide deeper insight into the relationship between their structural stability and aggregation propensity. Despite ongoing research, their behavior remains largely unpredictable, with tissue damage often detected only at advanced stages. It is estimated that between 25 and 75% of patients with multiple myeloma will develop kidney failure over the course of the disease (Yadav et al. 2015; Kundu et al. 2022). Moreover, approximately 25% of patients already show advanced kidney damage at the time of initial diagnosis (Bladé et al. 1998). To date, no confirmed correlation exists between the severity of tissue damage and the molecular properties of FLCs. However, pathogenesis is assumed to be influenced by protein-dependent factors intrinsic to monoclonal FLCs that predispose them to misfolding and aggregation (Basnayake et al. 2011). These factors may be determined by the individual amino acid sequence or may arise later through post-translational modifications and mutations that compromise structural integrity. Once initiated, the microenvironment—including the biochemical and physiological characteristics of the tissue, as well as the presence of specific interaction partners—may further promote aggregation (Bliznyukov et al. 2005). This complexity must be carefully considered when analysing FLCs.

To examine the effects of disulfide bond reduction on FLC species distribution and aggregation dynamics, nine purified FLC samples were incubated under reducing conditions for different periods of time. TCEP, which selectively and irreversibly reduces both inter- and intramolecular disulfide bonds within minutes (Burns et al. 1991), was used to induce destabilization. The reduction of disulfide bonds with TCEP is an established, but deliberately harsh, in vitro stress protocol for probing protein stability and triggering aggregation (Li et al. 2018; Sequeira et al. 2019; Melnikova et al. 2019; Džupponová and Žoldák 2023). Because this condition does not reproduce the complexity of physiological environments, the size, morphology, and surface chemistry of the resulting aggregates may differ from those that form naturally. Accordingly, the present data define one mechanistic limit—rapid aggregation after loss of disulfide bond stabilization—rather than a direct replica of in vivo aggregation pathway. The progression of changes in FLC species distribution following disulfide bond destabilization was monitored in a series of SV experiments. This approach enabled an analysis of structural stability and aggregation behavior as a function of incubation time in the presence of TCEP. Although the aggregates observed in this study were generated experimentally using reducing conditions, our use of the term ‘aggregation’ is not restricted to this specific pathway. Rather, it refers more generally to irreversible, misfolding-driven assemblies of potential pathological relevance. TCEP addition resulted in a time-dependent redistribution of molecular species in all samples, with disulfide bond reduction acting as a trigger for the observed aggregation process. Significant differences were observed among the samples in both the proportions of aggregates formed and the rates at which these species developed. In conclusion, after 15 h of incubation sample P001 exhibited the lowest aggregate proportion (~7%), while sample P020 demonstrated the highest level of aggregation (~73%). The remaining samples showed an average aggregate content of approximately 55% after the same incubation period. These findings indicate that the *λ*-FLC P001 demonstrates the highest stability, whereas P020 is the most susceptible to aggregation among *κ*-FLCs. However, this apparent relationship between aggregation stability and propensity does not correlate with patient clinical data, nor with the documented degree of renal damage at the time of sample collection (Sternke-Hoffmann et al. 2020). The Kidney Disease: Improving Global Outcomes (KDIGO) nomenclature of chronic kidney disease categorizes patients into five stages based on their glomerular filtration rate (GFR), ranging from normal kidney function (stage G1) to renal failure (Levey et al. 2020). Patient characteristics reveal that P020 shows only a mild decline in kidney function (G2), while patient P001 has progressed to moderate impairment (G3A). Similarly, patients P006 and P017 display distinct levels of kidney function despite comparable aggregation levels after 15 h of TCEP incubation. While patient P017 presents normal kidney function (G1), patient P006 show an advanced renal impairment (G4). Therefore, it must be assumed that the specific type of aggregate, rather than the overall quantity, may play an important role in tissue damage. Since protein concentration is a well-established factor in driving self-assembly, it is likely that distinct FLCs exhibit unique concentration thresholds where they achieve peak aggregation levels. Additionally, factors beyond the structural destabilization of FLCs—such as the microenvironment—likely contribute to the formation of nephrotoxic aggregates as the disease progresses (Bliznyukov et al. 2005).

The SV experiments demonstrated different levels of resistance to reducing conditions among the dimeric FLC species. In most samples, a substantial loss of dimer was observed within 1.25 h of TCEP incubation. However, samples P001, P004 and P020 showed greater resistance, with a pronounced dimer signal still present after 1.25 h of incubation. Notably, sample P001 maintained a dimer signal of approximately 64% of total sample even after 15 h of incubation. This suggests that TCEP-resistant dimers may exhibit sequence-specific features that contribute to a buried disulfide bond within the three-dimensional structure, limiting solvent accessibility and thereby preventing dissociation. Another possibility is that non-covalent interactions could stabilize the dimeric structure following disulfide bond reduction. However, this would be unexpected for sample P001, as covalent disulfide bonds are typically the primary stabilizing interactions facilitating dimer formation in *λ*-FLCs (Kaplan et al. 2011). No direct correlation was identified between the degree of dimerization and aggregation tendency. However, findings indicated that monomers dissociating from dimers lack stability in isolation and immediately transition into the aggregation pathway. It has been demonstrated that dimers formed under specific stress conditions exhibit structural differences from their native counterparts (Knight et al. 2022). Monomeric units within stress-induced dimers showed an increasingly non-native structural profile compared to native dimers. Consequently, it can be assumed that monomers resulting from forced dimer dissociation may also structurally differ from native monomers, potentially explaining the observed variation in stability. Given that the FLC samples analyzed are pathological, it should be considered that some dimers may inherently be stress-induced, having formed due to disease-associated alterations within the patients.

Previous studies on variable and constant domain fragments of FLCs have demonstrated that the reduction of disulfide bonds does not affect the secondary structure or folding state of the domains (Goto and Hamaguchi 1979; Frisch et al. 1996). However, after extended incubation with TCEP, a decrease of approximately 0.2 S in the *s*-value of the monomeric signal was detected in the *c*(*s*) distribution, suggesting an altered sedimentation behavior. This shift could reflect partial unfolding of the monomer upon the reduction with TCEP, as both the variable and constant domains of FLCs contain intramolecular disulfide bonds. Supporting this interpretation, CD spectra obtained before and after TCEP treatment revealed secondary structure changes, further implying structural differences. A possible connection between TCEP-induced secondary structure changes and FLC aggregation was observed by comparing sedimentation and CD spectroscopy data. The results indicate that an early loss of secondary structure following TCEP reduction may be associated with an increased aggregation tendency of FLCs. Prolonged TCEP incubation also resulted in the formation of low molecular weight fragments, with an increase in the corresponding signal at approximately 0.1 S observed following the addition of reducing agent. This increase tends to correlate with the duration of incubation. Therefore, it must be considered that the observed decrease in monomeric *s*-value may also be associated with a loss in molecular mass.

Although disulfide bonds have been shown to have only a minor influence on the overall structure of FLCs, they remain essential for their stability (Goto and Hamaguchi 1979). Studies using a variable domain fragment have demonstrated that the intramolecular disulfide bond contributes to folding stability by lowering the entropy of the unfolded state (Frisch et al. 1996). It is assumed that this decrease in entropy limits the conformational freedom of the unfolded state, thereby favouring the native folding of the domain. Consequently, while the disulfide bond is not strictly required for correct folding, it enhances stability and facilitates faster achievement of the native folding state (Frisch et al. 1996). The effect of disulfide bonds on the stability of the nine FLCs examined in this study was assessed through DSF. Measured melting temperatures of FLCs without TCEP addition aligned with previously determined values obtained using differential scanning calorimetry (DSC) and nanoDSF (Sternke-Hoffmann et al. 2023). Sample P006, which exhibited the lowest dimer proportion at approximately 10%, showed no change in melting temperature following disulfide bond reduction. In contrast, sample P001, consisting entirely of dimers, displayed a substantial decrease in melting temperature of nearly 10 °C upon disulfide bond reduction. The remaining samples also showed a correlation between the reduction-induced decrease in melting temperature and the degree of oligomerization, which was also expressed by the *s*_20,*w*_ of the total *c*(*s*) distribution. This trend is further supported by the response of FLCs to changes in ionic strength. Samples with a higher degree of oligomerization, such as P001, P004, and P020, exhibited more pronounced decreases in melting temperature upon addition of NaCl, both with and without TCEP. In contrast, monomeric or less oligomeric samples, including P006 and P017, remained largely unaffected, showing minimal or no changes in *T*_m_. These findings suggest that intermolecular interactions stabilizing oligomeric assemblies are particularly sensitive to electrostatic screening effects at higher ionic strength. The resulting destabilization likely lowers the energy barrier for thermal unfolding. This observation further reinforces the link between quaternary structural organization and thermal stability and supports the broader conclusion that FLC oligomerization modulates sensitivity to environmental stressors.

In addition to an increase in low molecular weight fragments with a *s*-value of approximately 0.1 S during TCEP incubation, another signal at around 1 S was detected in the samples P006, P016 and P017, suggesting further fragmentation. Notably, these three samples share the genetic origin from the germline sequence IGKV1-33, potentially indicating a sequence-specific predisposition to fragmentation (Sternke-Hoffmann et al. 2023). For the other FLCs, however, no correlation between germline sequence and species distribution was found. The underlying cause of the observed release of low molecular weight fragments as well as fragments associated with IGKV1-33 FLCs remains inconclusive. However, FLCs remain stable over extended incubation times in the absence of TCEP, with no fragmentation detected. Physiologically, fragment formation is often the result of proteolytic cleavage by endoproteases. Endoproteolysis, in turn, is influenced by the kinetic stability of dimeric structures, as the dimeric state protects FLCs from proteolytic cleavage (Morgan and Kelly 2016). While fragments from the variable domain usually predominate in pathological aggregates (Glenner et al. 1970), aggregates consisting primarily constant domain fragments have also been documented (Olsen et al. 1998). Unlike the variable domain, the constant domain appears to be able to resist further endoproteolytic cleavage (Solomon et al. 1998). Mass spectrometry analysis identified traces of various cathepsins across all nine patient samples (Dupré et al. 2021). These enzymes co-purified with FLCs from patients urine samples due to their similar molecular weight of 20 to 35 kDa (Turk 2001; Yuzhalin et al. 2018). However, as cathepsin levels were often near the detection threshold, their concentrations were insufficient for visualization in *c*(*s*) distributions or on SDS-PAGE. Given that samples are stable without TCEP addition, it can be assumed that disulfide bond reduction exposes previously inaccessible regions that are susceptible to cathepsin cleavage. Cathepsins show the highest activity in a reducing environment at a low pH of around 5 (Turk et al. 2012; Yadati et al. 2020). Whether cathepsins remain active under the experimental conditions used in this work and contribute to observed fragmentation is currently under further investigation. Breaks within the peptide backbone may occur in patients but only become apparent during experimental destabilization. Studies have shown that in addition to reducing disulfide bonds, TCEP itself can induce peptide bond cleavage (Liu et al. 2010). Under certain conditions, an unspecific side reaction can lead to the cleavage at cysteine residues, potentially causing TCEP-induced protein fragmentation, particularly during extended incubation periods. Given that fragmentation observed in the IGKV1-33 samples occur after prolonged TCEP incubation, this mechanism cannot be ruled out. This raises questions about specific characteristics in IGKV1-33 sequences that could underlie this distinctive fragmentation pattern. However, the evidence for two distinct fragmentation mechanisms suggests that identified fragments may result from an interplay between these processes.

## Conclusion

The purification of FLCs from patient urine represents a non-invasive sampling method, yielding high FLC concentrations with sufficient purity for detailed biophysical and biochemical analyses. In search for prognostic markers to predict renal complications in monoclonal gammopathies, such as multiple myeloma, this study emphasizes the critical role of disulfide bonds in preserving the structural integrity of FLCs and mitigating their tendency to form nephrotoxic aggregates. In summary, loss of disulfide bonds caused a redistribution of molecular species and aggregation as observed by SV-AUC after different TCEP-incubation periods. FLC dimers were differently resistant to dissociation, possibly due to non-covalent interactions or variations in cysteine accessibility to the reducing agent. Varying incubation times revealed that, in most cases, dimers do not dissociate into stable monomers but instead accumulate directly into higher-order oligomers and aggregates. Prolonged reducing conditions led to FLC degradation, as evidenced by an increase in low molecular weight fragments that were detectable at approximately 0.1 S but did not sediment adequately at 60,000 rpm. A simultaneous decrease in monomeric *s*-value with increasing TCEP incubation times suggested potential unfolding, further supported by CD spectroscopy, which revealed secondary structure changes. Notably, FLCs derived from the IGKV1-33 gene exhibited unique fragmentation behavior under reducing conditions, while FLCs from other germline sequences did not demonstrate a connection between germline origin and molecular properties. A correlation between the oligomerization state and the melting temperature shift upon TCEP treatment further highlights the critical role of disulfide bonds in stabilizing FLCs, particularly in promoting dimerization through covalent bonding. While the in vitro behavior of purified FLCs offers valuable insights, its correlation with in vivo behavior across diverse patient tissue environments remains uncertain, as no direct correlation to patient-specific clinical data was observed. It is important to acknowledge that the clinical data available in this study represent only a single time point corresponding to sample collection at initial diagnosis, leaving both the pathological changes preceding diagnosis and the subsequent disease progression unexamined. To address these limitations, future studies should prioritize longitudinal monitoring of FLC levels in both serum and urine over the course of disease progression, with particular attention to their changes in relation to renal damage. Numerous additional factors likely influence the pathological behavior of disease associated FLCs, potentially evolving alongside disease progression. Tracking changes in patient characteristics over time may improve the interpretability of biophysically determined FLC properties and their pathological roles within the clinical context. Rather than expanding the sample size, future studies could focus on a selected cohort of patients with periodic sampling throughout disease progression. This approach would enhance the precision and relevance of the findings, supporting validation of the in vitro characteristics of FLCs described here as potential prognostic markers.

## Supplementary Information

Below is the link to the electronic supplementary material.

Supplementary file1 (DOCX 326062 kb)

## Data Availability

All raw data are available from the authors upon request.
