# Supplementary material for: Tracking reduction-induced molecular changes in pathological free light chains by SV-AUC
Source: Eur Biophys J. 2025 Aug 5;54(6):365–83. doi: 10.1007/s00249-025-01788-2 (PMC12552258; doi:10.1007/s00249-025-01788-2)
Supplement: Supplementary file 1 — Supplementary file1 (DOCX 326062 kb) [file 249_2025_1788_MOESM1_ESM.docx]

Supplementary Material

Tracking reduction-induced molecular changes

in pathological free light chains by SV-AUC

Florian T. Tucholski^1^, Rebecca Sternke-Hoffmann^2^, Thomas Pauly^1,3^, Rasmus K. Norrild^4^, Amelie Boquoi^5,6^, Roland Fenk^5^, Luitgard Nagel^1,3^, Alexander K. Buell^4^, Rainer Haas^5^, Dieter Willbold^1,3^

^1^Institut für Physikalische Biologie, Heinrich Heine University Düsseldorf, Germany; ^2^PSI Center for Life Sciences, 5232 Villigen PSI, Switzerland; ^3^Institute of Biological Information Processing (IBI-7: Structural Biochemistry), Research Center Jülich, Jülich, Germany; ^4^Protein Biophysics Group, Department of Biotechnology and Biomedicine, Technical University of Denmark, Søltofts Plads, Building 227, Kgs. Lyngby, Denmark; ^5^Department of Hematology, Oncology and Clinical Oncology, Heinrich Heine University Düsseldorf, Germany ^6^Department of Hematology and Stem Cell Transplantation, West German Cancer Center and German Cancer Consortium, University Hospital Essen, University of Duisburg-Essen, Essen, Germany

Methods

**Calculations and data analysis**

**Table S1.** Fit parameters used to analyse SV-AUC sedimentation data.

| **Sample ID** | $\bar{\boldsymbol{v}}$ **[cm^3^ g^–1^]** |  | **Buffer solution** | **Density [g/cm^3^]** | **Viscosity [*P*]** |
| --- | --- | --- | --- | --- | --- |
| P001 | 0.726160 |  | 30 mM Tris-HCl, pH 7.4 | 1.0004 | 0.010124 |
| P004 | 0.726223 |  | + 0.1 M NaCl | 1.0045 | 0.010224 |
| P005 | 0.725521 |  | + 1 M NaCl | 1.0407 | 0.011091 |
| P006 | 0.726200 |  |  |  |  |
| P007 | 0.725071 |  |  |  |  |
| P013 | 0.726996 |  |  |  |  |
| P016 | 0.726208 |  |  |  |  |
| P017 | 0.724235 |  |  |  |  |
| P020 | 0.726701 |  |  |  |  |

Results

**Changes in FLC size distribution after disulfide bond reduction**

**Figure S1. Influence of disulfide bond reduction on the monomer-dimer distribution of FLCs.** The *c*(*s*) distributions of purified patient samples (**A**) P004, (**B**) P006, (**C**) P007, and (**D**) P016 are shown. Samples were prepared at a concentration of 35 µM in 30 mM Tris-HCl, pH 7.4 (black). To reduce disulfide bonds, 7 mM pH adjusted TCEP was added, followed by incubation at 37°C with shaking at 600 rpm for periods of 1.25 h (blue), 5 h (green), and 15 h (red). After incubation, 390 µL of each sample was transferred to the sample sector of standard aluminum double-sector cells, and sedimentation analysis was performed at 60,000 rpm and 20°C.

**Figure S2A. Sedimentation profiles of FLC samples.** Shown are the sedimentation profiles of patient samples (**A**) P001, (**B**) P004, (**C**) P005, (**D**) P006 and (**E**) P007, before and after TCEP treatment. A FLC concentration of 35 µM was measured in 30 mM Tris-HCl, pH 7.4. Disulfide bond destabilization was induced by the addition of 7 mM pH adjusted TCEP, followed by incubation at 37°C and 600 rpm shaking for 1.25 h, 5 h and 15 h. A total of 70 scans were measured per sample, with every third scan displayed for clarity.

**Figure S2B. Sedimentation profiles of FLC samples.** Shown are the sedimentation profiles of patient samples (**F**) P013, (**G**) P016, (**H**) P017 and (**I**) P020, before and after TCEP treatment. A FLC concentration of 35 µM was measured in 30 mM Tris-HCl, pH 7.4. Disulfide bond destabilization was induced by the addition of 7 mM pH adjusted TCEP, followed by incubation at 37°C and 600 rpm shaking for 1.25 h, 5 h and 15 h. A total of 70 scans were measured per sample, with every third scan displayed for clarity.

**Figure S3. Effect of varying the minimum sedimentation-coefficient limit (*s*_min_) and of applying Bayesian modification on the detection of low-*S* species in sample P001.** Plain maximum-entropy regularization was performed with *s*_min_ = 0 S (red), 0.05 S (black) and 0.1 S (blue); the same data were re-analyzed with Bayesian modification at *s*_min_ = 0 S (green). Half-peaks at *s*_min_ indicate that the lower boundary is constraining the fit, as described by Schuck (Schuck 2016): they grow taller as smin is lowered when baseline correlation is not suppressed. Setting s_min_ = 0 S and applying Bayesian modification removes this artifact and recovers the full peak that represents genuine low-*S* species (e.g., fragments or buffer components). The fragment peak at ~0.1 S is therefore a real, reproducible signal rather than a fitting artifact.

**TCEP-induced changes in secondary structure of FLCs**

**Figure S4. High tension (HT) voltage records for the CD spectra of all nine FLC samples before and after TCEP treatment.** For each sample, HT records are shown for the untreated state (black) and after incubation with 7 mM TCEP for 1.25 h (blue), 5 h (green) and 24 h (red) incubation time. Incubation was carried out with a sample concentration of 35 µM in 10 mM sodium phosphate buffer, pH 7.4 at 37°C. Aliquots were taken after specified time points and diluted to a final concentration of 9.23 µM for CD measurements.

**Table S2.** Position of zero-crossing of ellipticity (~200 – 210 nm) as a function of TCEP incubation time.

| **Patient sample** | **Zero-crossing**  **without TCEP**  **[nm]** | **Zero-crossing**  **after 1.25 h**  **[nm]** | **Zero-crossing**  **after 5 h**  **[nm]** | **Zero-crossing**  **after 15 h**  **[nm]** |
| --- | --- | --- | --- | --- |
| P001 | 207.00 | 206.90 | 206.40 | 204.50 |
| P004 | 207.00 | 206.70 | 198.00 | 199.70* |
| P005 | 205.10 | 200.20 | 196.00 | 197.70 |
| P006 | 204.40 | 203.30 | 195.00 | 195.00 |
| P007 | 206.00 | 199.40 | 198.90 | 200.00 |
| P013 | 207.00 | 205.40 | 200.90 | 198.00* |
| P016 | 205.70 | 202.20 | 203.90 | 195.80* |
| P017 | 206.00 | 198.70 | 196.60* | 200.00 |
| P020 | 206.50 | 201.50 | 197.40 | 201.90 |

*HT values above 700 V at wavelength below 200 nm render the data unreliable

**Modulating effects of the molecular species distribution of FLC**

**Figure S5. Effect of varying salt concentrations on the molecular species distribution of FLCs.** Shown are the *c*(*s*) distributions for samples (**A**) P006, (**B**) P007, (**C**) P017, and (**D**) P020 with the addition of 0.1 M (orange) or 1 M (red) NaCl. A 30 mM Tris-HCl buffer at pH 7.4 and a FLC concentration of 35 µM was used. To reach equilibrium, the samples were incubated for 24 h at 25°C prior to measurement. After incubation, 390 µL of each sample was transferred to the sample sector of standard aluminum double-sector cells, and sedimentation analysis was performed at 60,000 rpm and 20°C.

**Figure S6. Salt-induced shifts in weight-average *s*-values of FLCs.** Illustration of changes in weight-average *s*-values for samples P006, P007, P017, and P020, caused by the addition of 0.1 M (orange) or 1 M (red) NaCl. Corresponding weight-average *s*-values without NaCl addition are shown in black. The sedimentation range from 0 to 7 S was integrated to calculate the weight-average *s*-value, capturing the combined contribution of all species in this region and reflecting the overall distribution trend for each sample. Dashed lines labelled with 'M' and 'D' indicate *s*-values for individually identified monomers and dimers, representing their average *s*-value range across samples.

**Figure S7. Sedimentation profiles of FLC samples with addition of 0.1 M and 1 M NaCl.** The *c*(*s*) distributions of samples (**A**) P006, (**B**) P007, (**C**) P017 and (**D**) P020 are shown. A FLC concentration of 35 µM was measured prepared in 30 mM Tris-HCl buffer at pH 7.4, with the addition of 0.1 M or 1 M NaCl. To reach equilibrium, the samples were incubated for 24 h at 25°C prior to measurement. A total of 70 scans were measured per sample, with every third scan displayed for clarity.

**Figure S8. Sedimentation profiles of FLC samples at a concentration of 12.5 µM.** The *c*(*s*) distributions of samples (**A**) P007, (**B**) P013 and (**C**) P016 are shown. All FLC samples were prepared at a concentration of 12.5 µM to investigate the presence of a dynamic monomer-dimer equilibrium. Samples were measured in 30 mM Tris-HCl buffer at pH 7.4. A total of 70 scans were measured per sample, with every third scan displayed for clarity.

**Thermal stability of FLCs under reducing conditions**

**Figure S9. DSF data of all patient derived FLCs.** A sample concentration of 35 µM was prepared in 30 mM Tris-HCl, pH 7.4 (blue). To induce disulfide bond destabilization, 7 mM TCEP (red) were added in addition. Thermal denaturation was monitored using 10 µM SYPRO Orange in a total volume of 25 µL per well, measured in triplicate. Presented are the thermal denaturation profiles for all nine FLC samples. Elevated pre-transition fluorescence suggests that SYPRO Orange binds to native light chains, unfolded species, or other sample components, such as light chain fragments.

**Figure S10. DSF data of all patient derived FLCs with the addition of 0.1 M NaCl.** A sample concentration of 35 µM was prepared in 30 mM Tris-HCl, pH 7.4 with the addition of 0.1 M NaCl (blue). To induce disulfide bond destabilization, 7 mM TCEP (orange) were added in addition. Thermal denaturation was monitored using 10 µM SYPRO Orange in a total volume of 25 µL per well, measured in triplicate. The thermal denaturation profiles are presented alongside the corresponding negative derivative fluorescence-temperature plots (−d*F/*d*T*) for patient samples P001 (**A**), P004 (**B**), P005 (**C**), P006 (**D**), P007 (**E**), P013 (**F**), P016 (**G**), P017 (**H**) and P020 (**I**). Elevated pre-transition fluorescence suggests that SYPRO Orange binds to native light chains, unfolded species, or other sample components, such as light chain fragments.

**Figure S11. DSF data of all patient derived FLCs with the addition of 1 M NaCl.** A sample concentration of 35 µM was prepared in 30 mM Tris-HCl, pH 7.4 with the addition of 1 M NaCl (dark blue). To induce disulfide bond destabilization, 7 mM TCEP (dark red) were added in addition. Thermal denaturation was monitored using 10 µM SYPRO Orange in a total volume of 25 µL per well, measured in triplicate. The thermal denaturation profiles are presented alongside the corresponding negative derivative fluorescence-temperature plots (−d*F/*d*T*) for patient samples P001 (**A**), P004 (**B**), P005 (**C**), P006 (**D**), P007 (**E**), P013 (**F**), P016 (**G**), P017 (**H**) and P020 (**I**). Elevated pre-transition fluorescence suggests that SYPRO Orange binds to native light chains, unfolded species, or other sample components, such as light chain fragments.
